# Extra Supplement—The Nursing Mirror

**Published:** 1890-08-23

**Authors:** 


Being the Extra Nursing Supplement of "The Hospital" Newspaper.

All Contributions for this Supplement should be addressed to the Editor, The Hospital, 140, Strand, London, W.C., and should have the word "Nursing" plainly written in left-hand top corner of the envelope.

### En Passant.

ANOTHER INSTITUTION.—Mrs. Fitch, who has for some time carried on the St. Vanda's Home at Bromley, has started an institution for trained nurses at Surbiton. Mrs. Fitch was trained at Westminster and Queen Charlottes, and learnt massage under Mrs. Creighton Hale. The nurses at St. Vandas' seem to be well cared for.

ZENANA MEDICAL COLLEGE.—This college has lately secured a tremendous amount of patronage, so following the stream of fashion, we made our way to 58, St. George's Road, to have a look at it. Miss Robinson, who has so often and so kindly forwarded us news of the work, was not in, but Miss Libbis, the matron, was quite ready to give all information. The college is quite full; each cubicle has its occupant, but even with a crush there is only room for fifteen students. A session is just over, and those who have completed their two years' training are dispersing to far-off lands; two to the interior of China, two to Africa, and one, a member of the Plymouth Brethren, is going to India on her own account. An Armenian girl, now going through her training, will carry her knowledge back to Turkey for the use of the women in the harem. There is a splendid lecture-room and a good medical library at the college; the whole place seems bright and cheerful, and filled with earnest life and purpose.

THE AUSTRALIAN COMMISSION.—For the present our inquiry into metropolitan charities is in abeyance, but news still comes from Australia of the inquiry going on there The chief evidence with regard to nursing has been that of Dr. Balls Headley, of the Alfred Hospital: "Women are better fitted by their constitution for nursing than men. The training provided at the Alfred Hospital is excellent, but the hours are too long. Nurses should not be on duty for longer than ten hours a-day, and they should not be asked to do scrubbing or coarse work. The Government ought to have some influence in the management of hospitals, and should be represented on the committees of management, or on a general board of supervision." Dr. Barrett, secretary of the Medical Society of Victoria, said that nurses should be trained; their hours were too long. Dr. J. E. Nield, for thirty-seven years in practice in Melbourne, said he was glad to know that nurses were now being more systematically trained. Care should be taken, however, that they did not supersede their functions. He had heard, for instance, of trained nurses carrying about with them hypodermic syringes to give patients a dose of morphia when they could not sleep. Dr. J. P. Ryan said he did not think any *trained* nurse would take it upon herself to give a dose of morphia. Mr. T. N. Fitzgerald, surgeon, representative of the British Medical Association, said he thought 10 hours a-day long enough for a nurse to work. Mr. W. H. Gibson, secretary to the Women's Hospital, and for 17 years cashier at Manchester Royal Infirmary, said there were 21 nurses in the Women's Hospital, and their hours were from half-past six a m., to half-past eight p.m., one day, and on the following day from half-past six a.m., to half-past six p m., and so on, alternately. He thought the hours were long, but the duties were not heavy. He had a complaint some time ago from one of the nurses as to the length of the day- and the head nurse had objected to going on duty after eight o'clock at night. The general social status of the nurses at the hospital was good. No one can read the report of the Melbourne inquiry, and not notice, so far as nursing goes, how frivolous in comparison are the points brought forward in England.

NURSES AT ROME.—Four of the American nurses of St Paul's Home, Rome, have returned to New York, and will be replaced by winter with four more nurses. The conditions are board and lodging and 15 dollars a month, the nurse promising to stay 18 months and serve as a private nurse, especially amongst English speaking people, on all parts of the continent. Any surplus above the expenses of the home which is earned, is given to the American mission in Rome.

MALE NURSES.—It is amusing to note in the fifth annual report of the Hamilton Association the different treatment accorded to the male nurse. To begin with he has to deposit caution money on entering, so that if he misbehaves this is forfeited; again he is subject to fines of from 5 to 40 shillings, and in 16 cases these fines were enforced last year. On the other hand the male nurse only trains for one year, and is paid while he trains; he is then sent to private cases. "The highest amount deducted is 15 per cent., but on some cases deductions of only 10 and 5 per cent. are made. The enrolled nurses receive the wages they earn—less these deductions respectively—and are not paid an annual pittance and provided with board and lodging when not employed." It is rather a bitter fling at we women nurses that "annual pittance." But apparently the male nurses have to be kept in order by much more stringent rules than is necessary with the gentler sex. Here is the hand of friendship to you, brethren of the forceps, and may the Hamilton Association continue to flourish.

NURSE'S HOME, JOHANNESBURG.—The following is an extract from Miss Mollett's letter to the local paper which we mentioned last week:—"The home is conducted on strictly unsectarian lines, and the committee is entirely undenominational. For the future four beds for the in-patients will be reserved for those who are unable to pay the fees, and the number will be increased, should the generosity of the public enable us to do so. Practically a number of patients have already been nursed free of charge, as the amount owing for nursing fees since February 1st of this year is over £200, the greater part of which it is very unlikely will ever be paid. It is my intention to keep a nurse at the Home to undertake district work among the poor, free of all charges. Maternity cases are also now received at the Home, entirely separate from the other cases." The income from subscriptions last year was £415, of which £10 was from Mr. Roberts, champion billiard player. During the year 127 patients were received, and nurses were sent out to 72 cases. Besides the five nurses Miss Mollett took out with her the staff has been re-inforced by two nurses from the Kimberley Hospital, and three probationers. The nurses Miss Mollett took with her were: Miss Kirkpatrick (Sister). Where trained, Charing Cross Hospital, London; Soho Hospital for Women. Posts held, Sister, Charing Cross Hospital; Sister, Leicester Hospital. Miss Hickman (Nurse), Nottingham Hospital. Post held, Sister, Cramarthen Hospital. Miss Welby (Nurse). Where trained, Nottingham Hospital; Alexandra Children's Hospital, London. Post held, Head Nurse, Royal Cornwall Hospital. Miss Sheeman (Nurse). Where trained, Rhyl Children's Hospital; Children's Hospital, Great Ormond Street, London; Guy's Hospital, London. Miss Rose Blennerhasset (District Nurse). Where trained, Salop Infirmary; Lambeth General Lying-In, London. Posts held, Superintendent, Cardiff Union Hospital (London Obstetrical Soc. Diploma).

### Sketches of Insanity.

By Francis H. Walmsley, M.D. (Senior Assistant Medical Officer of Leavesden Asylum).

### VII.—DEMENTIA.

The normal life of an individual, his whole organisation—intellectual, emotional, and moral—is kept in a condition of freshness and integrity only by the incessant activity of his memory and intelligence, and by the conscious perception of the things of the external world; it is the failure of the power of responding to the impressions received from without that is so striking a feature in the demented. In this defect of sensitive reaction we have a criterion which indicates to the observer the secret dilapidations which have occurred in the sphere of mental activity. A man in a state of dementia is deprived of advantages which he formerly enjoyed, he was a rich man who has become poor, he is dethroned from a higher to a lower phase of consciousness, he has become less impressionable, less curious as to knowledge and feeling, he lends but an inattentive ear to the things of the external world, he lives in himself and upon remembrances of the past; days pass away, the world goes by, events succeed around him, he no longer pays attention, and the progressive indifference and invading apathy which manifest themselves in him, attest the gradual exhaustion of the vital forces of his mental activity.

Dementia generally comes on gradually, the faculties, both moral and intellectual, decay one by one, the deterioration of mental function extends from failing memory and slight confusion of thought to utter fatuity. In the earlier stages it is remarked that the individual is not the same he once was; he has lost his freshness and spontaneity, there is a weakening of the higher and holier impulses, and of the ideas and sentiments that rendered him interesting and sociable; he is content to pass his life within the limits of a contracted sphere of thought and action. He may appear mentally healthy to those who see him for the first time, but essentially and to his friends there is a profound change, the decadence of the moral sensibility follows that of the intelligence step by step. In a man intellectually degraded we can only count upon a low morality. As the disease progresses the memory of recent events becomes impaired, there is dulness of perception and apprehension; an inability to fix the attention, to follow out any train of thought, and to carry on consecutively a conversation of any length; the patient displays a lack of initiative, never originates anything, and is utterly helpless in action, hence the transaction of business requiring sustained attention becomes impossible, passing events produce little, if any, impression, while the past is remembered with tolerable freshness, and the patient can reason fairly correctly with regard to it, when he cannot clearly appropriate and rightly estimate the present; old memories remain the freshest and most vivid, while recent facts—impressions which occur at the very moment—are unperceived and treated as if they did not exist; a task interrupted is forgotten, incidents of the day, an order received, or a resolution made, these are soon effaced.

Many dements on the day after their admission into an asylum insist that they have been there a year, five years, ten years. They have only the vaguest recollection of leaving their homes and their families; they can tell neither the day of the week nor the name of the month; things heard five minutes since are forgotten; they cannot recollect what they had for breakfast or dinner; have lost all idea of their own identity, and have absolutely forgotten their own names; they frequently take persons about them for those who are absent or dead; they lavish caresses and words of endearment on dolls and other articles, which they evidently regard as their children. Many are able to arise, dress themselves, take their meals regularly, play at cards or other games, frequently with remarkable skill. They can still employ themselves mechanically—men in writing and women in knitting or sewing; the routine of daily life and habits long contracted are the last to be abolished. The new perishes and the old endures; the recollections of infancy are the last to disappear, and even in an advanced stage of the malady experiences and songs of childhood often return.

The next phase is one of complete incomprehension; memory, reason, the power of attention, and of perceiving the true relations of things are entirely lost; the talk is made up of fragmentary and incoherent rambling; the logical and associating bonds are altogether wanting; sometimes the same word or phrase is repeated for many hours together; persons so affected know their attendants, and recognize their friends and relatives, but they seldom display signs of emotion on seeing them. Nearly all dements retain some tendency to slight attacks of maniacal excitement. During these explosive outbursts acts of violence may be committed, or there may be a manifestation of destructive impulses of an automatic character, patients tearing up their clothing or bedding, and destroying everything within their reach; in some, amid the general wreck of mind, their delusions retain a more or less powerful hold of them, and determine their actions. One whose singular movements seem unaccountable is busy spinning threads out of sunbeams, while another continues the most violent movement of his arms in order to prevent the motion of the universe or of his own blood from coming to a standstill. Others are fixed in one position, believing themselves made of glass or wood; the chief and most common delusions of senile dements relate to their property.

In some patients the muscular force often remains intact and displays itself in perpetual activity, in jumping and running to and fro, walking round in a circle, rocking backwards and forwards, dancing, singing, shouting, or in talking and muttering incessantly; many manifest a strong disposition to collect all kinds of useless articles, which are hoarded up as if they were of great value; other patients again sit silent and tranquil for weeks, months, or even years, giving expression to a low monotonous moaning, or fitfully uttering a few sounds; others remain couched in uneasy posture, manifesting not the slightest interest or concern in what is passing on around them. In the last stage the unfortunate sufferer lives, and that is all, being scarcely conscious of existence; he is reduced to vegetative life, eating, drinking, and sleeping, and becomes more and more a child, caring no longer for those who day after day care for and tend him.

Dements constitute two-thirds of our insane population, forty out of every hundred of all new cases of insanity pass sooner or later into dementia.

*(To be continued.)*

### A Prayer for the Hospital Ward.

Command Thy blessing, Gracious Lord, on all admitted here,

The maimed, the sick, the suffering, help each their cross to bear.

Grant patient resignation, submission to Thy will,

Knowing Thou workest all for good Thy promise to fulfil.

Give strength for every trial, new hope and peace impart,

To soothe the sad and weary and cheer the fainting heart.

Recall back to their memory how Thy dear life was spent

In patient sympathy for all who to the Saviour went;

And send Thy angel messenger, that blessed boon bestow,

And give to Thy beloved sleep—the balm for human woe.

O let Thy loving voice be heard within each sufferer's breast,

With welcome words—"Come, weary one, and I will give you rest."

And if by Thy good providence they are relieved from pain,

Blessed by the treatment here received, to health restored again,

May gratitude inspire each heart to go where duty calls,

Remembering their Father's care within these hallowed walls.

Bless those who here devote their lives to work and wait on Thee,

Fulfil Thy gracious "Forasmuch ye did it unto me,"

And prosper Thine own handiwork, let theirs more prosperous be,

Thine be the glory, Thine the praise, throughout eternity.

### The Training of Nurses in France.

The third volume of the *Manuel Pratique* is the thickest of the series, and deals very fully with surgical instruments and dressings. The illustrations are numerous; all the chief instruments, specimens of splints, and baths and syringes, are given, and greatly help to the clear understanding of the letterpress. Strange that in England our nursing manuals are so poorly illustrated; save in the case of bandaging, wood-cuts are almost unknown. The first chapter very wisely deals with the necessity of pure air for surgical cases, whether treated in the wards or private rooms; the second chapter deals with beds and bed-making. Many of the directions in this volume are already out of date, for English nurses at least; for instance, the proper preparation for a fractured femur bed is said to be to put a wooden plank under the top mattress. The directions for carrying or moving wounded persons are wise, in so far as they beg the nurse to take firm hold of her charge without signs of fear, not to timidly grasp the sleeve of the night-gown; but they make too much of the necessity of two persons for lifting and carrying. There is so much knack and knowledge about this, that an experienced nurse can do without effort what a strong man would stagger under. Nor is it necessary to lift a patient for the changing of sheets; we know a better plan than that in our English hospitals, and the St. John's Ambulance Association has spread the knowledge of how to roll and shift a sheet far and wide. All the details regarding antiseptics are given, and the sponges are treated in the wet method and kept in antiseptic solution, not in the dry method advocated by Lawson Tait. Poultices, fomentations, ice bags, liniments, injections, &c., are all described, with the advantage of the illustrations before mentioned. There is not much that is new to be learnt from these chapters, and it may amuse our readers to know that massage is dismissed in a couple of pages. So much for fashion in therapeutics! The chapter on fractures is interesting, and the splints described are complicated, and we should fancy not cleanly for hospital wards. The simple rules, "In doing an ordinary dressing a nurse should never cause a wound to bleed," and "A nurse should never touch a wound either with her fingers or her instruments," are excellent. For washing a wound a nurse is bidden to let the water run over it, and, if necessary, clean the edges with a piece of lint moist with antiseptic lotion. The cleanliness of fingers or instruments can never be quite depended on. A whole article is devoted further on in this manual to the cleanliness of the hands, when the use of soap and the nail brush before, as well as after, every dressing is forcibly insisted on. The subject of hæmorrhage and how it can be arrested, the use of leeches, dry and wet cupping, and even scarifying, are dealt with; and an illustrated chapter is given to the consideration of clinical thermometers. The scale used is Centigrade, normal point being 36·8 deg. and fever point 40 deg. The time necessary to take a temperature in the axilla, or in the palm of the hand is stated to be from ten to fifteen minutes. Very rarely the temperature may exceed 42 deg., or descend lower than 36 deg., but such temperatures are regarded as abnormal. With regard to the injection of morphia the directions are good, but the evils of the morphia habit, and the power of the nurse in discouraging it are not touched on. If all we hear is true, Paris is the stronghold of this baneful habit, and surely a word of warning to nurses would have been wise. The somnolence and sickness likely to follow a morphia injection are spoken about, also the risk of the needle breaking beneath the skin. The duties of the nurse during the visit of the physician are accurately detailed, also her part in preparing a patient for chloroform. "In every case a patient who has been under an anæsthetic should be carefully watched until he has completely regained his senses." This volume concludes with the duties of the nurse towards the dead; this is not so full as we could wish; the subject is a disagreeable one, but should not be slurred over.

*(To be continued.)*

### A Daring Outrage.

Last week a case of more than usual interest to readers of The Hospital was decided at West Ham by the magistrate, Mr. Baggallay. It appeared from information tendered in Court, by Mr. Herbert William Whyte, resident medical officer of the new hospital in the Albert Docks, that on the night of Saturday, the 9th inst., a man, supposed to be a quarter master on an Atlantic cattle ship, effected an entrance into the hospital through a first-floor window. He had previously taken the precaution to remove his boots, and to leave them outside the building. He then made his way into a bedroom in which two nurses were in bed asleep. On being aroused by a call from her sister nurse, one of these young women, with great courage and presence of mind, sprang out of bed and pushed the intruder out of the room. Mr. Whyte, hearing a call from the night nurse, went to the foot of the stairs and saw the man descending. He promptly sent for the police who took the man into custody. There was ample evidence of the man's designs in entering the hospital. On Monday, the 11th inst., Mr. Whyte, Mr. Adams, the assistant secretary, and the nurses attended at the West Ham Police Court to prosecute. The prisoner pleaded guilty, but stated that he had been "silly drunk," and was very sorry for what had occurred. The magistrate thereupon, and without further inquiry, discharged him with a fine of five shillings. The Committee of the Seamen's Hospital very properly took a different view of the matter, and instructed counsel to apply for a warrant for the apprehension of the offender on a charge of burglary with intent to commit a rape. The application was accordingly made on Wednesday, the 13th, by Mr. R. W. Cracroft, of the Midland circuit. But Mr. Baggallay, declining to go back upon his own decision, held that on the facts shown in the information, there was no evidence of the felonious intent to commit a rape, and expressed the opinion that the Burglary Act was not intended to cover cases of immorality. He accordingly refused process either by way of warrant or summons; but expressed his agreement with the observation of counsel, that young women in the position of hospital nurses especially required and deserved the protection of the law against outrages of such a character as had been committed. It is to be presumed that the magistrate administered the law as it stands. But if so, it is very plain that the law is in immediate need of amendment if hospital nurses are to be adequately protected in their much-exposed and dangerous positions.

### Everybody's Opinion.

[*Correspondence on all subjects is invited, but we cannot in any way be responsible for the opinions expressed by our correspondents. No communications can be entertained if the name and address of the correspondent is not given, or unless one side of the paper only be written on.*]

### WORKHOUSE NURSES.

Miss Louisa Twining writes:—If the "discouraged" nurse had carefully read the article I think she would have seen that the description applied entirely to country workhouses, and not to those exceptionally good and wholly different institutions,—the metropolitan infirmaries — of which she is a member, and for which good nurses are easily obtained. For those other sick wards, where we rightly speak of neglect, we ask that nurses will come forward and throw themselves into the breach, even though it may be in solitary places. The hon. secretary of the Workhouse Infirmary Nursing Association writes that she has numerous applications, which she is unable to supply for lack of duly trained and suitable women, who naturally prefer to join those institutions where their comfort is well cared for, as is truly described by the writer. (P.S.—May I add that since writing the article I have heard from an inmate of one of these country unions, saying "If you had had the misfortune to have been an inmate of one of these places you could not have explained the situation more fully.")

### PARIS INSTITUTIONS.

"Dr. S." writes: The chief institution in Paris is the Levick Institution of English Trained Nurses, 34, Rue de Prony. None but thoroughly trained nurses are employed, and it is well administered by Mrs. Levick—a woman of great ability. These are the nurses which the English (and even foreign) doctors are glad to get. The demand is so great that Mrs. Levick has difficulty in meeting it; particularly was this the case in the influenza epidemic. The whole thing is a worthy representative of English institutions.

### INTEMPERATE NURSES.

"Secretary" writes: Regarding the question raised by a lady superintendent on the classification of intemperate nurses, I should be glad to know what are the actual powers given to employers of speaking or writing the truth? The recent decision in the case of the Mayor of Newcastle and Mr. H. M. Stanley's valet, in which damages were awarded against the Mayor for giving his opinion on the valet's honesty, has puzzled many persons who consider it a duty to warn employers against an intemperate or otherwise undesirable employé. Until the decision in this case, I was under the impression that a *bonâ fide* communication (in which malice was unproved) was considered privileged. I think that inquiries as to character would be much more truthfully and fully answered if the law of libel was clearly understood. In many cases there is an evasion, questions being passed over which, if answered, would ensure a real knowledge of the candidate's character. It should be noted that it is in the interests of good nurses that the black sheep in the profession should be known, and should not be employed. Many of the numerous complaints against trained nurses proceed from the fact that, especially in private nursing, women are passed on from families to institutions, and *vice versâ*, with most insufficient references. Testimonials from doctors, and one or two from matrons of ancient date, are not enough to ensure a knowledge of the qualities desirable in the nurse we may be sending to a responsible position. To guard against recommending an intemperate and otherwise objectionable nurse, a recent reference to matrons or to employers who have known the applicant some time are needful. The case of nurses who have been "nursing a relative," and have no recent references, require to be carefully investigated. In some cases that "relative" has been found to be a home for inebriates, and "good people," who have been helping to pay the expenses of reform in such a home, occasionally allow their consciences to sleep in suppressing the real facts in such cases, although they give their names as references! This they call "giving a chance to the poor woman," but is it not a culpably unfair action to the employers, the patients, and to good nurses? Private nursing homes, district nursing committees, and occasionally, but in a less degree, provincial hospitals suffer so severely from insufficient or untruthful testimonials and references that I shall be glad if The Hospital can help us to raise the whole standard by (1) a paper on testimonials and the law of libel, and (2) by receiving and classifying suggestions which might, later on, lead to an informal conference.

### Death in Our Ranks.

The death is announced, on the 10th inst., at Bath, of Miss Margaret Taylor, formerly Superintendent of the Bath Home for Trained Nurses.

We record with regret the death of Mrs. Margaret Naylor, aged 75, for many years matron of the Manchester and Salford Lock Hospital.

### Notes and Queries.

To Correspondents.—1. Questions may be written on post-cards. 2. Advertisements in disguise are inadmissible. 3. In answering a query please quote the number. 4. If a private answer is desired, a stamped addressed envelope must be forwarded.

Notice.—We must really ask our correspondents to be more particular in enclosing name and address. Constantly we receive queries or letters with no name attached.

### Answers.

*Harley Street Home.*—"One who has Taken an Interest for Years in the Harley Street Home" must be perfectly aware that no credit is ever attached to anonymous contributions.

(32) *Microscopes.*—I should strongly advise Sister Rachel, unless she has a good sum of money at her disposal, not to get a "stand" from any of the well-known microscope makers. I got an excellent unnamed one from the Stores, firm tripod, revolving stage, for a very moderate sum, because it was not stamped by a well-known maker's name. Moreover, if expense is to be considered, I advise the purchase of a microscope without fine adjustment, as this can be added at any time at no greater cost than if the microscope had been originally purchased with it. As regards objectives, if for botany two will be required, 1 inch and ½ inch power; if histology is to be studied, ½ inch and ¼ inch will be the most useful. Messrs. Baker and Sons, of Holborn, are the agents for Zeirs' objectives, which are cheaper than English makes, and are often preferred to the latter. The Civil Service Supply Association, 136, Queen Victoria Street, E.C., will also procure Zeirs' objectives and allow discount. Messrs. Baker have second-hand English and German objectives in stock, and will send a list on application, and if you are in London would let you have the less expensive ones (which are also the very high powers) on approval. I myself got two very good objectives by Ross in this way, and if you are lucky you may get very good second-hand ones cheaper even than Zeirs'. As regards books, "Strasburger's Botany" gives very full directions about the microscope; for histology Harris and Power's book is very good and inexpensive.—*Somerset.*

### Vacancies.

The following vacancies are announced:

**Nurses.**

Barnhill Poorhouse, Glasgow; £45.

Bromley and Surbiton Institutions.

Burton-on-Trent Infirmary; £25

Burton-on-Trent Institution; £25.

Bishop's Stortford Institution; £25.

Cornelia Hospital, Poole.

Cambridge Home.

Eastern Fever Hospital, Homerton; £30.

Eastern Fever Hospital, Homerton (Assistant); £22.

Haywood Hospital, Burslem; £23.

Hanover Institute, W.; £25.

Kingston-upon-Hull Workhouse Hospital (Night); £30.

Lock Hospital, Harrow Road.

Merthyr Hospital; £20.

Monkwearmouth and Southwick Hospital; £25.

Mercers' Hospital, Dublin; £20.

Royal Albert Hospital, Devonport; £25.

Swansea and South Wales Institute; £25.

Sheffield Union; £23.

Suffolk Hospital, Bury St. Edmunds; £25.

St. George's Home, Sheffield.

Swinton Industrial School; £20.

St Saviour's Infirmary, East Dulwich; £20.

St. Mark's Hospital for Fistula, E.C.; £30.

Victoria Home, Bournemouth; £25.

Wolverhampton Hospital.

**Probationers.**

Boston Hospital.

Birmingham and Midland Skin and Lock Hospital; £10;

Bristol Hospital for Children.

City Hospital, Coventry.

Tunbridge Wells Hospital.

### Amusements and Relaxation.

### SECOND QUARTERLY WORD COMPETITION.

Commenced July 5th, 1890, ends Sept. 27th, 1890.

Three prizes of 15s., 10s., 5s., will be given for the largest number of words derived from the words set for dissection.

Proper names, abbreviations, foreign words, words of less than four letters, and repetitions are barred; plurals, and past and present participles of verbs, are allowed. Nuttall's *Standard* dictionary only to be used.

N.B.—Word dissections must be sent in WEEKLY not later than the first post on Thursday to the *Prize Editor*, 140, Strand, W.C., arranged alphabetically, with correct total affixed.

The word for dissection for this, the EIGHTH week of the quarter, being

"HEATHER."

| Names. | Aug. 14th. | Totals. | Names. | Aug. 14th. | Totals. |
|---|---|---|---|---|---|
| Dulcamara | 112 | 400 | Jenny Wren | 106 | 382 |
| Tinie | 120 | 419 | Rhoda | — | 243 |
| Henri | 120 | 395 | Annabel | — | 17 |
| Agamemnon | 123 | 426 | Spare Moments | — | 19 |
| Patience | 121 | 419 | Qu'appelle | 105 | 388 |
| Brisbane | 97 | 332 | Nurse | — | 29 |
| Nurse Hilda | 119 | 399 | A. B. C. | 69 | 251 |
| Lightowlers | 127 | 421 | Brickbat | — | 77 |

### Notice to Correspondents.

N.B.—Each paper must be signed by the author with his or her real name and address. A *nom de plume* may be added if the writer does not desire to be referred to by us by his real name. In the case of all prize-winners, however, the real name and address will be published.

Competitors can enter for *all* quarterly competitions, but no competitor may take more than *one first prize* during the year.

